# Forgotten in the tropics: research on *Culex* mosquitoes is overshadowed in malaria and dengue-endemic regions

**DOI:** 10.1186/s13071-026-07309-0

**Published:** 2026-04-17

**Authors:** Abdisalam A. Abdi, Ryan Almeida, Trevor Harris, Tereza Magalhaes, Jose G. Juarez, Gabriel L. Hamer

**Affiliations:** 1 Department of Entomology, Texas A&M University, College Station, TX USA; 2 Department of Statistics, University of Connecticut, Storrs, CT 06269 USA; 3 College of Veterinary Medicine and Biomedical Sciences, Texas A&M University, College Station, TX USA

**Keywords:** *Culex* mosquitoes, *Aedes* mosquitoes, *Anopheles* mosquitoes, Endemicity, Malaria, Dengue, Zoonotic arboviruses, Systematic review, Vector-borne diseases, Neglected vectors

## Abstract

**Background:**

In many countries where mosquito-borne diseases such as malaria and dengue are endemic, the research community focuses on studying the mosquito vectors of these diseases in the *Anopheles* and *Aedes* genera, respectively. In these settings, other mosquito taxa, including *Culex* spp. and associated pathogens, appear less frequently in published studies. Although the field widely recognizes that several mosquito taxa and pathogen systems are understudied in several regions, few studies have quantified these patterns.

**Methods:**

We conducted a systematic literature review of mosquito-related studies published in 2010 and 2020 to test the hypothesis that the proportion of mosquito publications on *Culex* spp. would be higher in countries that are non-endemic for malaria and dengue. Studies were identified through PubMed and Web of Science using “Country + mosquito” keyword searches, screened by inclusion/exclusion criteria, and categorized by endemicity (malaria-endemic, dengue-endemic, both, and non-endemic). We summarized mosquito genera per study and compared their reporting frequencies using generalized linear mixed models (beta–binomial likelihood) adjusted for year and GDP per capita.

**Results:**

After screening 10,834 unique publications, 1,389 met inclusion criteria. The average number of mosquito genera reported per study was significantly higher for non-endemic countries compared with countries endemic for malaria and dengue. Publications including data on *Culex* spp. mosquitoes were significantly higher for non-endemic countries (64.5%) compared with malaria endemic (30.2%) and dengue endemic (34.2%) countries. Between 2010 and 2020, reporting of *Aedes* spp. increased, whereas reporting of *Anopheles* decreased, consistent with changing global research emphasis over the decade, including the 2015–2017 Zika emergence and continued dengue expansion.

**Conclusions:**

These results indicate that the presence of human-amplified mosquito-borne pathogens (e.g., human malaria and dengue) is associated with lower reporting of *Culex* in the published field-collection literature and with comparatively less published attention to *Culex*-associated zoonotic pathogens. A step to help resolve this neglect is for researchers to include additional mosquito community data when publishing malaria and dengue vector studies. These findings can help the research and public health community to allocate attention on multiple vector-borne disease threats, proportional to the respective human health burden.

**Graphical Abstract:**

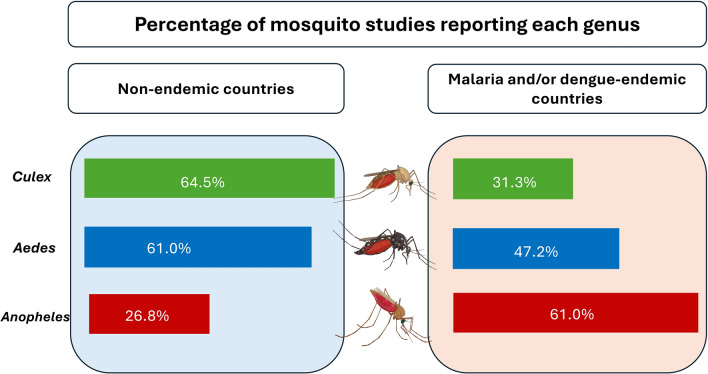

**Supplementary Information:**

The online version contains supplementary material available at 10.1186/s13071-026-07309-0.

## Background

Mosquitoes are considered the most important arthropod vectors of pathogens that impact public health [1, 2]. While they are regarded as the “world’s deadliest animals,” this fame is driven by mosquito-borne diseases such as malaria and dengue [3]. In 2023, malaria caused an estimated 263 million cases and 597,000 deaths [4]. Meanwhile, dengue virus places nearly half of the world’s population at risk of infection, most of which live in tropical and subtropical regions, especially in urban and semi-urban settings[5–7]. Each year, an estimated 100–400 million dengue infections occur worldwide, resulting in about 100 million symptomatic cases and approximately 21,000 deaths [8, 9]. These infection risks underscore the public health threats malaria and dengue pose in regions where they are endemic. They also highlight the importance of understanding the pathogen and mosquito vectors that maintain their transmission to guide effective control strategies. Nonetheless, understanding vectors beyond those associated with these two main diseases is critical for developing comprehensive vector control programs that can better prepare communities against emerging or re-emerging arbovirus threats.

In parallel, many pathogens are transmitted by *Culex* spp. mosquitoes which impose non-trivial burdens. Japanese encephalitis virus (JEV; *Orthoflavivirus japonicum*) is a leading cause of viral encephalitis in Asia; the World Health Organization (WHO) summarizes an estimated ~100,000 clinical cases annually, and global modeling work has estimated on the order of ~100,000 cases and ~25,000 deaths in 2015. In addition, ~1.54 billion people living in areas suitable for sustained JEV transmission (≈38% of > 4 billion residents of Japanese encephalitis (JE)-endemic countries) [10–12]. West Nile virus (WNV; *Orthoflavivirus nilense*) is estimated to cause between 6 and 16 million infections annually worldwide, though precise global figures remain uncertain owing to variable surveillance capacity across regions [13] In the USA, where surveillance is well-established, WNV has caused an estimated 7 million infections since 1999, with over 60,992 reported cases and more than 3,134 deaths reported from 1999 to 2024 [14, 15]. Global seroprevalence is estimated at 3.6%, with case-fatality rates of approximately 10% among neuroinvasive cases, though 80% of infections are asymptomatic [13, 16–18]. Lymphatic filariasis remains widespread, affecting 120 million people globally, and in 2024 an estimated ~657 million people remains threatened and required preventive chemotherapy [19].

Human malaria is caused by multiple species of parasites with *Plasmodium falciparum* being the dominant species globally, especially in Africa, while *Plasmodium vivax* is more common in Central and South America and Southeast Asia [4, 20, 21]. Multiple *Anopheles* spp. mosquitos transmit these *Plasmodium* spp. parasites such as *Anopheles gambiae*, *An. arabiensis*, and *An. funestus* in Africa and *An. darlingi* in South America [22–25]. *Aedes*-borne viruses are transmitted by *Aedes* (*Stegomyia*) mosquitoes with the primary vector globally being *Aedes aegypti* and a secondary vector being *Aedes albopictus* [26, 27]. Both human malaria and *Aedes*-borne viruses exist primarily in what are considered human-amplified cycles or sometimes urban cycles, where *Anopheles* spp. or *Aedes* spp. mosquito vectors transmit the pathogens among humans [28, 29]. For these pathogens, humans are considered the amplification host and develop a parasitemia or viremia capable of infecting mosquitoes, thus completing the urban transmission cycle. For the purpose of this study, we refer to pathogens in urban cycles with only human amplifiers, and no wild or domestic animal amplifiers, as “human-amplified mosquito-borne pathogens”.

The majority of mosquito-borne pathogens do not exist in urban cycles and instead are zoonotic and exist in sylvatic or enzootic transmission cycles. These pathogens are amplified in domestic or wild animals, which completes the enzootic cycle. Bridge vector mosquito species are ones that feed on parasitic or viremic domestic or wild hosts, become infected, and then subsequently feed on humans [30]. For these pathogens that exist in enzootic cycles, humans are generally not capable of developing a parasitemia or viremia and are thus considered “dead-end hosts” with respect to the pathogen [31–33]. Zoonotic mosquito-borne pathogens that exist in these enzootic cycles include WNV, Usutu virus (*Orthoflavivirus usutuense*), St. Louis encephalitis virus (SLEV; *Orthoflavivirus louisense*), JEV, Venezuelan equine encephalitis virus (VEEV; *Alphavirus venezuelan*), and eastern equine encephalitis virus (EEEV; *Alphavirus eastern*) [34]. These sylvatic mosquito-borne pathogens are transmitted by many different mosquito taxa globally, especially members of the *Culex* genus. *Culex* spp. mosquitoes have a cosmopolitan distribution and often have broad (generalist) host feeding patterns, including the feeding on species of birds and other wild animals that serve as amplification hosts [35, 36]. *Culex* spp. mosquitoes are also competent for a wide range of zoonotic pathogens [37–39].

A pattern that is recognized by the field but poorly supported or quantified with empirical data is that when a region is endemic for a human-amplified mosquito-borne disease such as malaria or dengue, these systems dominate research and public health attention [40]. For example, many infectious diseases, including those that are vector-borne, present with similar symptoms as malaria and dengue. In resource-poor settings, symptomatic patients are often assumed to have malaria or dengue when, in reality, this may not always be the case. Consequently, reporting biases develop when differential diagnoses are based on symptoms and not confirmed by PCR or other confirmatory tests [41–44]. For instance, in Brazil, SLEV was detected in patients initially diagnosed with dengue, showing how *Culex*-borne infections can be missed [45, 46]. Similar diagnostic challenges have been documented in malaria-endemic regions. For example, according to a synthesis by Oidtman et al. (2021), a study from India found that fewer than 6% of individuals presumptively treated for malaria actually tested positive, while approximately 25% were confirmed to have dengue instead [47]. Consistent with these patterns, an investigation from West Sudan documented undetected circulation of major arboviruses (including dengue and yellow fever) among malaria-negative febrile patients and attributed underrecognition to limited laboratory capacity and reliance on clinical diagnosis [48].

Likewise, in the presence of endemic human-amplified mosquito-borne pathogens, mosquito vector research and surveillance are focused on the specific vectors of those pathogens such as *Anopheles* spp. or *Ae. aegypti* mosquitoes. These biases lead to many of the other mosquito taxa, including those that are pathogen vectors, being neglected [40]. Several studies allude to this situation, including a systematic review of mosquito species distribution modeling that revealed that research in malaria-endemic regions predominantly focuses on *Anopheles* species, while studies in dengue-endemic areas concentrate on *Aedes* mosquitoes [49–51]. This selective focus has resulted in significant knowledge gaps regarding other mosquito genera, particularly in regions with rich mosquito diversity. This lack of knowledge of vectors beyond the dominant human-amplified systems can limit preparedness for emerging and re-emerging mosquito-borne threats transmitted by *Culex* and other mosquitoes responsible for the transmission of zoonotic pathogens [52]. For example, WNV is maintained in enzootic cycles and transmitted primarily by *Culex* mosquitoes; although WNV has been detected in 39 African countries (many malaria-endemic), genomic surveillance is available from only 16 countries, highlighting major gaps in molecular surveillance and, by extension, gaps in the vector-focused data needed to understand transmission. In addition, WNV is often misdiagnosed as malaria or classified as “undifferentiated febrile illness” when laboratory tests are negative [53–55]. These examples reinforce the value of broad mosquito surveillance beyond disease-specific programs focused narrowly on primary vectors.

The objective of this study was to conduct a systematic literature review comparing field-based studies of mosquitoes in countries that are endemic for human-amplified mosquito-borne pathogens and those countries that are not. We tested the hypothesis that countries endemic for malaria and dengue disproportionately concentrate their research efforts and reporting on *Anopheles* and *Aedes* mosquitoes, neglecting other important mosquito genera and the pathogens they transmit. These data will help identify knowledge gaps and allow research communities and public health authorities to balance attention on the multiple vector-borne disease threats to animals and humans.

## Methods

### Search strategy

We conducted a systematic literature review by searching the primary peer-reviewed literature indexed in Web of Science and PubMed, using keyword searches for “Country name + Mosquito” (e.g., Argentina + mosquito) for the years 2010 and 2020. Our searches were limited to peer-reviewed publications indexed in these databases; gray literature (government reports, theses, conference abstracts, unpublished datasets) was not systematically searched. We extracted publications on different mosquito species and mosquito-borne zoonotic pathogens across different countries. We focused on 165 countries classified as endemic for at least one of two human amplified mosquito-borne diseases: malaria (*n* = 83) and dengue (*n* = 82), with co-endemic for both defined as the overlap of these lists (*n* = 33), based on endemicity definitions published by the US Centers for Disease Control and Prevention (CDC) and the World Health Organization (WHO) (see Additional file 1: Sheet S1) [56–58]. These endemicity classifications reflect national-level presence/absence and do not capture within-country heterogeneity or transmission intensity. Additionally, 42 countries were included as non-endemic. These countries were selected on the basis of CDC and WHO classifications indicating no evidence of frequent, continuous, sporadic, or uncertain transmission for either dengue or malaria [57, 59].

This resulted in a total of 348 country-specific keyword searches (country + mosquito) per year across both databases (696 total across 2010 and 2020). We selected 2010 and 2020 as two cross-sectional sampling years to compare research reporting patterns at two time points. Owing to the large volume of resulting literature, often in the thousands per year, and the need for manual screening, we limited our search to these 2 years on the basis of available resources. When performing the searches in Web of Science and PubMed, we selected the “all fields” option, which allowed the query to search across all searchable fields within each record. This approach ensured that our search terms were identified regardless of where they appeared in the publication metadata or content. The full database search query templates and search settings for PubMed and Web of Science (including field selection and year limits) are provided in (Additional file 1: Supplementary Sheet S3). Because our inclusion criteria required field-based mosquito collections, we expect this strategy to capture most eligible mosquito-focused studies, although several records may still be missed owing to variability in indexing or reporting. Authors AAA and RA performed the literature searches, identified papers to include and exclude, and extracted data out of papers meeting inclusion criteria. For papers with uncertainty about the inclusion criteria, they discussed with GLH.

### Study selection and screening

For each country, we recorded the search conducted and documented the total number of publications returned. Each publication was reviewed to determine whether it met our predefined inclusion and exclusion criteria. Publications were included only if they met all inclusion criteria and were excluded if they met any of the exclusion criteria.

Inclusion criteria were: (1) study reporting field-based data collections of adult mosquitoes or immature mosquitoes (e.g., larvae, pupae, and eggs), (2) studies on all mosquito taxa, whether vector or non-vector, (3) primary studies reporting empirical data (with methods, results, etc.) from the country, and (4) studies in which a clearly described, representative field collection of mosquitoes (adult or immature mosquitoes (F0) generation) were collected from the field to be tested in the lab.

Exclusion criteria included: (1) studies conducted in the lab with no field-associated data, (2) studies that focused only on human samples and did not include mosquitoes, (3) studies based on a human questionnaire, (4) studies conducting mathematical models with no unpublished empirical data, and (5) studies in which only a small number of adult or immature mosquitoes were collected inconsistently or opportunistically with no standardized sampling design.

Studies reporting multiple mosquito genera were recorded for all genera present, such that individual studies could contribute to multiple genus categories simultaneously. For example, a study collecting both *Anopheles* and *Culex* mosquitoes was counted in both the *Anopheles* and *Culex* categories.

### Statistical analysis

We extracted all mosquito genera reported from each publication and summarized the proportions of studies that reported each genus. We analyzed reporting at the genus level rather than species level because many included studies identified mosquitoes only to genus, making consistent species-level synthesis infeasible across the global dataset. Our objective was to quantify publication reporting patterns (as a proxy for research attention) rather than species-specific vector importance or disease risk. Our outcome reflects genus reporting in publications and should not be interpreted as a direct measure of vector abundance, competence, or local awareness. To assess differences across endemicity groups, we focused on the three most frequently reported mosquito genera: *Anopheles*, *Aedes*, and *Culex*. Countries were categorized into four mutually exclusive endemicity statuses (malaria-endemic, dengue-endemic, both endemic, and non-endemic). Because our review sampled two calendar years (2010 and 2020), and multiple studies could occur within a country each year, we then aggregated records by genus, country, and year. For each combination, we counted the number of studies reporting that genus and the total number of studies. In addition to the models, we counted the number of mosquito genera reported per study and summarized this as mean ± standard deviation (SD) by endemicity category. The mean ± SD represents the average count of distinct genera across individual studies within each category, with SD indicating study-level variability in genus diversity.

We fit separate generalized linear mixed models (GLMMs) for each genus to test whether endemicity predicted whether a study reported that genus (present versus absent) while adjusting for calendar year (2020 versus 2010) and for economic context using standardized log gross domestic product (GDP) per capita. GLMMs were chosen because they handle unbalanced data and account for clustering of observations within countries [60] via their random effect terms. The response for each country–year was the number of studies reporting the genus (successes) out of the total number of studies (denominator).

We evaluated three possible likelihoods for the GLMM. First, a Poisson likelihood with a log offset for the total number of studies was found to be unsuitable because the responses are binomial counts bounded by the country–year total, and Poisson assumes unbounded counts with equal mean and variance (equidispersion). Negative-binomial likelihoods (also with an offset) relax equidispersion but still treat the numerator as unbounded and showed inferior fit reflected in higher AIC values and non-uniform residuals or overdispersion detected in DHARMa, indicating that the model did not capture variability well relative to binomial-scale models. We therefore adopted a beta–binomial likelihood with a logit link, which directly models the number of studies reporting the genus out of a fixed total and accounts for extra-binomial variation (over- or underdispersion) by allowing the success probability to vary across heterogeneous country–years [61, 62]. Models were fit with the glmmTMB package in R (version 4.5.1) [63].

We fit separate global models for each of the three genera (*Anopheles*, *Aedes*, and *Culex*) that included endemicity category (4-level factor: malaria-endemic, dengue-endemic, both endemic, or non-endemic), year (2020 versus 2010), and standardized log GDP per capita as fixed effects. For *Culex* and *Aedes*, we also included a random intercept for country to account for repeated measures across the two years. The analogous *Anopheles* model with a country random intercept showed convergence and identifiability problems (non-positive-definite Hessian) with non-estimable standard errors; following a prespecified identifiability fallback, we fit the same fixed effects without a country random intercept. For interpretation, we used genus-specific endemicity baselines: non-endemic for *Culex*, dengue-endemic for *Aedes*, and malaria-endemic for *Anopheles*.

Inference emphasized within-genus contrasts among endemicity levels. We reported adjusted odds ratios (aORs) and model-adjusted probabilities based on marginal means (emmeans) [64], using Holm’s procedure to control the family-wise error rate across the three planned contrasts per genus [65]. Model adequacy was assessed with dispersion checks from the performance package [66] and DHARMa simulation-based residual diagnostics (uniformity, dispersion, and zero-inflation; 500 simulations)[67]. We verified optimizer convergence, finite standard errors, and Hessian status from model summaries. Data wrangling used tidyverse and janitor; country codes were assigned via countrycode [68], and GDP data were obtained from WDI [69].

All statistical analyses and data visualizations were conducted in R (version 4.5.1) Additional packages used included sf, readxl, and ggplot2 for data visualization [70–73]. Statistical significance was defined as two-sided *P* < 0.05. Country boundaries were sourced from Natural Earth (public domain) and accessed via the rnaturalearth/rnaturalearthdata packages [74].

## Results

**Fig. 1:**
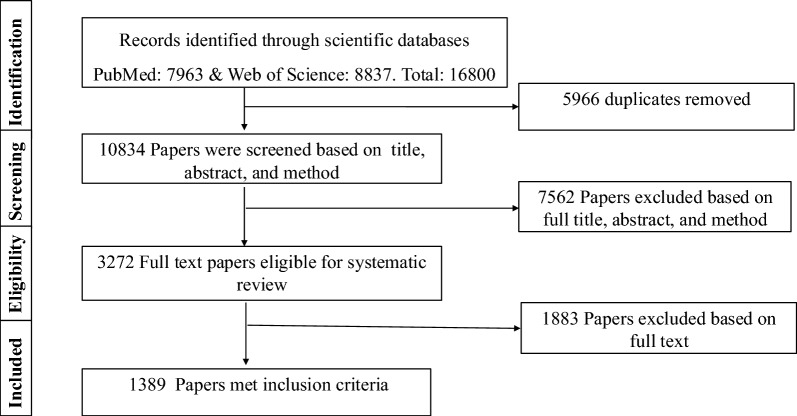
Study selection process for the systematic review; outlines the literature screening and selection process used in the systematic review years 2010 and 2020. It shows the number of articles identified, duplicates removed, records screened, full-text articles reviewed, and the final number of studies included (*n* = 1,389)

A total of 16,800 articles were identified through database searches. Of these, 5,966 duplicates were removed. In total, 10,834 articles were screened. On the basis of the title, abstract, and methods, 7,562 were excluded as they did not meet our study criteria. A total of 3,272 full-text records were reviewed, and of these, 1,883 were excluded on the basis of the inclusion and exclusion criteria (see Methods). Therefore, 1,389 articles published in 2010 (*N* = 487) and 2020 (*N* = 902) met our inclusion criteria for this systematic review (Fig. 1) (see Additional file 1: Supplementary Sheet S2).

### Geographic distribution of studies

**Fig. 2:**
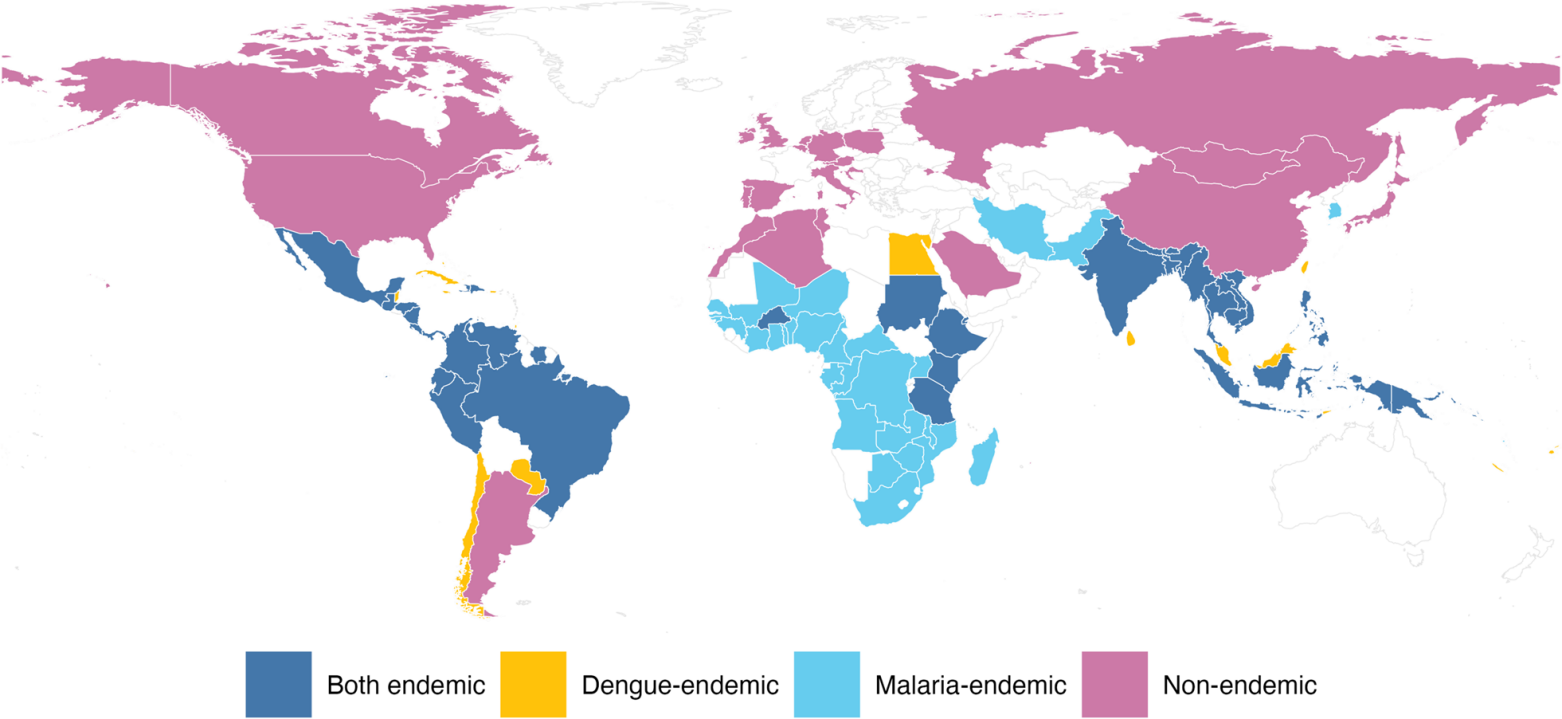
Endemicity of countries represented in included studies (2010 and 2020). Countries with at least one included study are shaded by their endemicity category, defined a priori from WHO/CDC lists: both (malaria and dengue endemic for both; dark blue), dengue-only (yellow), malaria-only (light blue), or non-endemic (magenta), using classifications from S1 (WHO/CDC lists) [57, 59]. Countries without included studies are shown as outlines only (see Additional file 2: Supplementary Table 2). Maps were generated in R using sf and ggplot2, with basemap and country boundaries from Natural Earth

Of the 1,389 included articles, studies were conducted across 118 different countries, including 36 in malaria–dengue (both)-endemic, 31 malaria-endemic, 20 dengue-endemic, and 31 non-endemic countries. The most frequently represented countries with mosquito publications meeting inclusion and exclusion criteria were the USA (*n* = 178 studies), Brazil (*n* = 123), and India (*n* = 74) (see Additional file 2: Supplementary Table 2) (Fig. 2).

### Mosquito genera across all studies

**Fig. 3:**
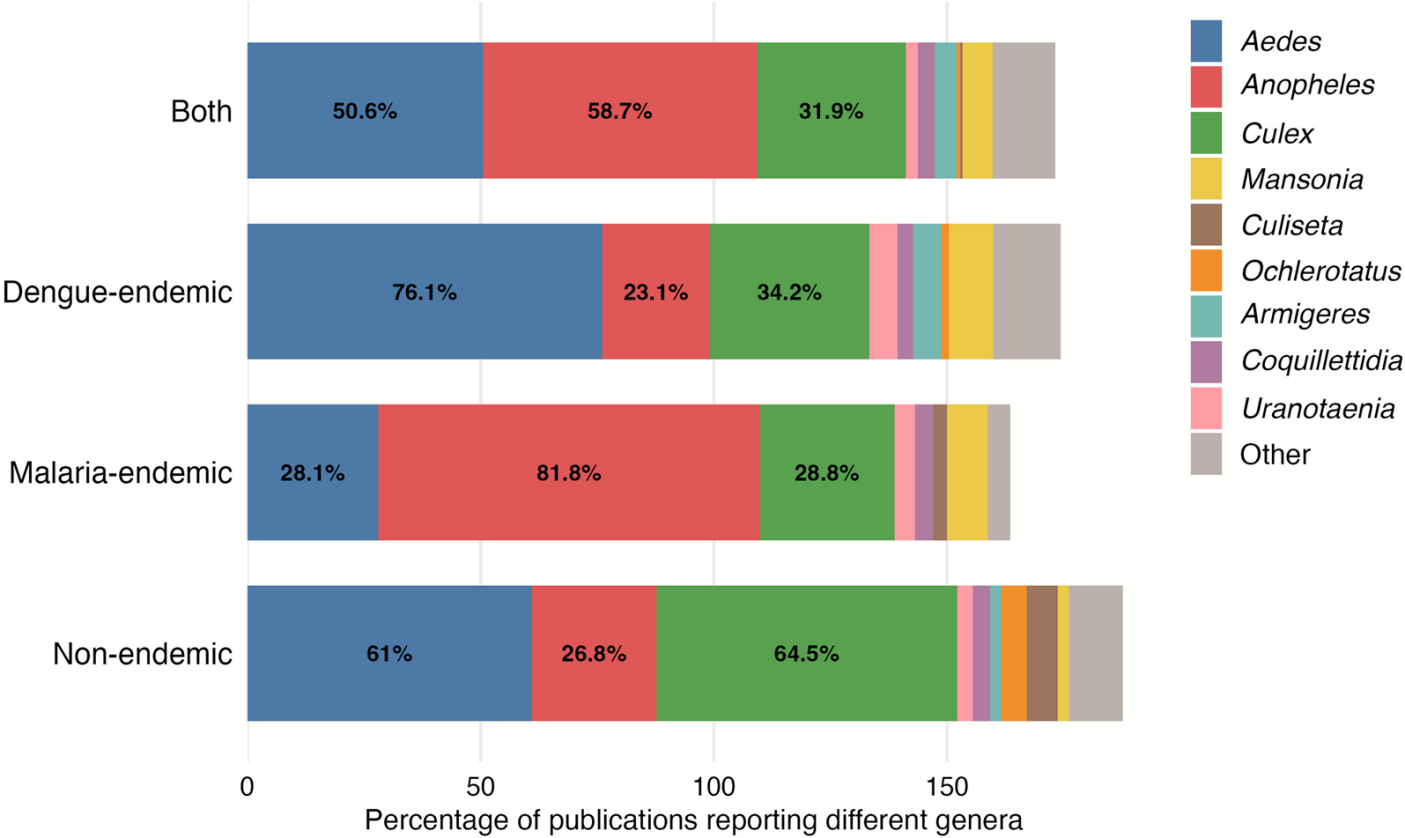
Percentage of publications reporting different mosquito genera by endemicity category. Bars are 100% stacked within endemicity categories; in-bar labels show study counts (*n*). Colors highlight *Aedes* (blue), *Anopheles* (red), and *Culex* (green); the remaining top ten genera are shown individually, and “Other” combines 20 additional low-frequency genera (see Additional file 2: Supplementary Table 1 for the complete genus list and study frequencies)

Across all included studies, the most frequently reported mosquito genera was *Aedes*, with 51.7% (*n* = 718) of publications including this genus. This was followed by *Anopheles* (49.7%, *n* = 691) and *Culex* (42.2%, *n* = 586). Less frequently reported genera included *Mansonia* (5.8%, *n* = 81), *Coquillettidia* (3.7%, *n* = 52), *Uranotaenia* (3.5%, *n* = 48), *Armigeres* (3.1%, *n* = 43), *Culiseta* (3.0%, *n* = 41), *Psorophora* (2.7%, *n* = 38), *Ochlerotatus* (2.2%, *n* = 31), and *Toxorhynchites* (1.7%, *n* = 23), *Haemagogus *(1.4, *n* = 19),* Wyeomyia *(1.2, *n* = 17), other genera, such as *Orthopodomyia*, *Lutzia*,* Mimomyia*, *Sabethes*, *Aedeomyia*, *Malaya*, *Tripteroides*, *Deinocerites*, *Heizmannia*, *Topomyia*, *Shannoniana*, and *Udaya,* were reported in 15 or fewer instances each, contributing ≤ 1.1% individually to the total genus mentions (see Additional file 2: Table 1) (Fig. 3).

The average number of genera per study was 1.88 ± 1.57 SD in non-endemic countries, 1.74 ± 1.64 in malaria-endemic countries, 1.74 ± 1.61 in dengue-endemic countries, and 1.68 ± 1.36 in countries endemic both malaria and dengue.

### Study-level genus inclusion

In malaria-endemic countries, *Anopheles* was reported most often at 75.6% (233/308), followed by *Aedes* at 34.1% (105/308), and *Culex* at 30.2% (93/308). In dengue-endemic countries, *Aedes* predominated at 76.1% (89/117), with *Culex* at 34.2% (40/117) and *Anopheles* at a less frequent 23.1% (27/117). In non-endemic countries, *Culex* was reported 64.5% (294/456), followed by *Aedes* at 61.0% (278/456), and *Anopheles* at 26.8% (122/456). In countries endemic for both malaria and dengue, *Anopheles* appeared in 60.8% of studies (≈309/508), *Aedes* in 48.4% (246/508), and *Culex* in 31.3% (159/508) (Fig. 3).

Model-based comparisons: the final beta–binomial models retained all fixed effects from the global model. For *Culex* and *Aedes*, the country random intercept was retained. For *Anopheles*, the country random effect showed convergence and identifiability problems (non-positive-definite Hessian); therefore, the final model included only fixed effects. All final models showed strong endemicity-related differences. For *Culex*, odds of being reported were significantly lower in all endemic settings compared with non-endemic countries: dengue-endemic aOR 0.33 (95% CI 0.15–0.73; Holm *P* = 0.0022), malaria-endemic 0.38 (0.17–0.86; *P* = 0.0052), and both-endemic 0.40 (0.19–0.83; *P* = 0.0052). The corresponding model-adjusted probabilities of reporting *Culex* were 56.2% for non-endemic, 29.9% for dengue-endemic, 32.8% for malaria-endemic, and 33.7% for both-endemic settings. For *Aedes* (reference = dengue-endemic), countries that were non-endemic (aOR 0.29, 0.11–0.80; Holm *P* = 0.0099) and malaria-endemic (0.34, 0.12–0.99; *P* = 0.031) showed lower odds of reporting *Aedes* than dengue-endemic countries, whereas the both-endemic contrast was not significant (0.52, 0.19–1.43; *P* = 0.123); model-adjusted probabilities were 66.7% (dengue-endemic), 37.0% (non-endemic), 40.5% (malaria-endemic), and 51.1% (both-endemic). For *Anopheles* (reference = malaria-endemic), odds of reporting were lower in non-endemic (aOR 0.39, 0.17–0.91; Holm *P* = 0.0159) and dengue-endemic (0.20, 0.08–0.50; *P* = 0.0001) countries, with a borderline difference for both-endemic (0.64, 0.36–1.12; *P* = 0.0558); model-adjusted probabilities were 71.2% (malaria-endemic), 49.2% (non-endemic), 33.3% (dengue-endemic), and 61.1% (both-endemic).

**Covariates.** After adjustment for endemicity, 2020 (versus 2010) was associated with higher *Aedes* reporting (aOR = 1.58, 95% CI 1.10–2.29), no clear change for *Culex* (0.83, 0.59–1.15), and lower *Anopheles* reporting (0.42, 0.29–0.61) Standardized log GDP per capita was also associated with genus reporting: higher GDP was associated with higher reporting of *Culex* (aOR ≈ 1.39 per SD; *P* = 0.023) and *Aedes* (aOR≈1.88 per SD; *P* < 0.001) and lower reporting of *Anopheles* (aOR ≈ 0.52 per SD; *P* < 0.001).

## Discussion

Our systematic review supports a long-recognized but underquantified trend: in countries where malaria or dengue is endemic, mosquito vector research report disproportionately on the primary human-amplified genera, *Anopheles* and *Aedes,* while data on *Culex* are underreported. Our results show that in malaria-endemic regions, *Anopheles* was represented in three quarters of studies (75.6%), consistent with its exclusive role in malaria transmission. While *Aedes* mosquitoes were the most frequently studied in dengue-endemic settings (76.1%) and remained highly represented in non-endemic regions (61.0%), consistent with their growing global significance as vectors of dengue, Zika, chikungunya, and yellow fever viruses. Given substantial disease-burdens for *Culex*-borne pathogens including JEV, lymphatic filariasis, and WNV, these patterns indicate a disproportionate underreporting of *Culex* in malaria- and dengue-endemic countries. We recognize that prioritizing malaria and dengue vectors in endemic settings is a rational public health response given limited resources and high disease burden. Our findings do not imply that malaria or dengue vector research should be reduced; rather, they highlight comparatively lower representation of *Culex* in published field-collection literature. Integrating surveillance on multiple mosquito taxa, including *Culex*, into existing malaria/dengue platforms may improve preparedness for zoonotic threats without detracting from core disease-control priorities.

The disproportionate representation of specific mosquito taxa in published field studies from malaria and dengue endemic settings may correspond with lower reporting of vectors of zoonotic pathogens. For example, many of the countries in this review currently classified as non-endemic for human-amplified mosquito-borne pathogens have established populations of *Anopheles* spp. and *Aedes* spp. mosquito competent for malaria and dengue, respectively. In addition, many of these countries were previously endemic for malaria and dengue, but owing to vector management and reduced mosquito-human contact, these human-amplified pathogens have been eliminated [75]. This was the case in the USA, where intense dengue epidemics once threatened many city centers throughout the eastern states, as far north as Philadelphia [76, 77]. In addition, the USA had widespread malaria transmission in the southeastern states up until the 1950 s [78]. Once human-amplified pathogens are eliminated as a concern, research appears to shift to focusing on the remaining public health threat from zoonotic pathogen and associated vectors, which likely contributes to the higher reporting of *Culex* in non-endemic countries. However, this does not mean that *Culex* and zoonotic pathogens are not present and causing human disease in countries that are endemic for malaria and dengue. The true public health burden caused by zoonotic mosquito-borne diseases is therefore difficult to assess in countries endemic for malaria and dengue [54, 79, 80].

*Culex* spp. mosquitoes are among the most geographically widespread mosquito species worldwide [81–83], transmitting several pathogens that affect not only humans but also livestock and wildlife, including WNV, JEV, SLEV, WEEV, EEEV, Usutu virus, Rift Valley fever virus, lymphatic filariasis, and avian malaria, contributing to significant ecological and economic impacts [84–92]. The outbreaks of *Culex-*borne diseases are becoming more frequent, coinciding with rising temperatures, climate variability, and changing land use patterns, all of which are expanding suitable habitats for these mosquitoes [38, 93]. As a result, they are expected to remain critical vectors of emerging and re-emerging pathogens [94–98].

Despite this, the role of *Culex* mosquitoes in pathogen transmission has been relatively neglected in public health discussions as shown by these results. This oversight could lead to underestimation of the health and economic burdens imposed by *Culex*-borne pathogens, contributing to knowledge gaps that may limit evidence-informed control and preparedness [99]. In several regions, the circulation of viruses transmitted by *Culex* spp. may be underestimated or go undetected. For example, a study conducted by Mencattelli et al. [54] highlighted several critical gaps in WNV surveillance and understanding across Africa. Their review found that multiple WNV lineages, specifically lineages 1, 2, and 8, are circulating on the continent, with numerous competent vector species, primarily from the *Culex* genus. However, they noted a shortage of vector competence studies for many mosquito species found naturally infected with WNV and widespread evidence of WNV circulation in humans, animals, and vectors across at least 28 countries. The study also highlighted that the epidemiological status of WNV remains largely unknown in 19 countries in Africa. Another study in Cameroon by Nchoutpouen et al. [100], similarly indicated that *Culex* spp. are widespread across the country and responsible for a high burden of nuisance in most urban settings; however, they remain less studied than *Anopheles* mosquitoes. Additionally, the detection of SLEV in cases initially suspected as dengue during an outbreak in Brazil underscores the potential for undetected circulation of other *Culex* spp. transmitted viruses [46].

Our findings align with longstanding pattern in the *Culex* spp. research literature. For example, Farajollahi et al. [35], in their review of the *Culex pipiens* complex, pointed out that the majority of host-feeding and vector competence studies had been conducted in North America, specifically the USA. Similarly, Griep et al. [101] synthesized 109 *Culex* bloodmeal studies across 30 countries and found pronounced geographic bias by biogeographical realm: the Nearctic (North America) contributed 54 studies (49.5%), whereas the Afrotropical realm (sub-Saharan Africa) contributed only 7 (6.4%). Tropical regions were also sparsely represented overall, including the Indomalayan realm (South and Southeast Asia; 4 studies, 3.7%) and the Neotropical realm (Central and South America; 16 studies, 14.7%). This imbalance is notable because many tropical settings have high *Culex* diversity and substantial burden from *Culex*-borne pathogens. Consequently, despite having a cosmopolitan distribution, we mainly understand the bionomics of members of the *Culex* genus on the basis of work mostly done in countries non-endemic for human-amplified mosquito-borne pathogens, which occur mainly in temperate regions of the world. As a result, many aspects of *Culex* related to host-feeding patterns, vector competence, and other critical factors from many parts of the world, especially the tropics, where *Culex* transmit pathogens resulting in human disease, remain unknown. Our findings also have implications for equity in research capacity. The concentration of detailed *Culex* bionomics and blood-feeding evidence in higher-income settings suggests that endemic regions may be undersupported in surveillance infrastructure, laboratory capacity, and training needed to characterize zoonotic arboviruses and their vectors. Strengthening local capacity, potentially by integrating *Culex* collections and testing into existing malaria/dengue platforms, may help close these knowledge gaps without diverting resources from primary vector control priorities. Although we highlight *Culex* as a prominent example, other less frequently reported genera (e.g., *Mansonia*, *Coquillettidia*, etc.) may also be underrepresented and warrant attention where they contribute to pathogen transmission or spillover to humans or animals.

We detected a temporal shift in the study of mosquitoes between 2010 and 2020, showing that publications on *Aedes* spp. mosquitoes increased in both endemic and non-endemic regions, consistent with heightened research activity following the 2014—2017 Zika pandemic and sustained dengue expansion [102–104]. In contrast, *Culex* representation remained consistently low across both time points, underscoring its persistent neglect despite ecological and epidemiological significance. The research on *Anopheles* spp. had a slight decrease over this time period, which could be due to slow reductions in the burden of malaria, which decreased from 896,000 deaths in 2000 to 627,000 in 2020 [105, 106]. As the Zika virus pandemic emerged, which was followed by an increase in funding for *Aedes* spp. mosquitoes [107], many research teams that previously studied *Anopheles* could have shifted to studying *Aedes*, which could be another mechanism for this shift over time. Because we did not systematically extract funding sources, we cannot determine whether these 2010 and 2020 differences reflect shifts in funding priorities, local research needs, or both.

This study was limited by several aspects. Nontarget genera may be underreported even when collected; therefore, lower reporting should be interpreted as lower representation in published literature rather than an absence of the data. Our experience while working with research teams focused on dengue and malaria is that *Culex* mosquitoes are observed or collected, but those data are not included in publications. This means that researchers doing the field work observe high *Culex* abundance, including in the indoor environment, but when these data are not published, the broader research community is unable to have this knowledge. While extracting out the taxa of mosquitoes studied in each of the 1,389 publications at the genera level, we did not track species level data. This is most problematic for *Aedes* as this is the mosquito genera with the highest taxonomic richness, including many species that are nuisance species and not known to be important vectors of pathogens. Although the vast majority of the studies from dengue-endemic countries reporting *Aedes* spp. mosquito data were on *Ae. aegypti* specifically, we do not have the exact breakdown at the species level. Another limitation is that this study only conducted the literature review for the years 2010 and 2020. Each year required 414 separate keyword searches, resulting in thousands of papers manually screened. Including additional years or decades could have captured additional patterns. For example, the global spread of WNV increased starting in 1999 when introduced to the Americas for the first time, with resulting spread throughout the Americas over the next several years [108]. This spread of WNV would have increased the research focused on *Culex* spp. mosquitoes, although the presence of SLEV in many of these same regions [109] would have already warranted a focus on *Culex* mosquitoes. In addition, endemicity was assigned at the country level on the basis of CDC/WHO classifications, which cannot capture within-country heterogeneity or transmission intensity and may misclassify settings, especially for large countries or those that stretch a long latitudinal gradient. Such misclassification would be expected to add noise and attenuate observed associations rather than generate spurious differences. Ecological and climatic context may also confound observed associations, because malaria- and dengue-endemic countries are restricted to tropical and subtropical regions, while *Culex*-associated zoonotic mosquito-borne pathogens exist from tropical to temperate regions [110]. Therefore, differences in *Culex* reporting may reflect both ecological contexts of where mosquito-borne pathogens circulate as well as research prioritization patterns, even though important *Culex*-borne pathogens also occur in tropical regions.

## Conclusions

Overall, our findings highlight an imbalance in scientific literature that aligns with dominant, disease-specific public-health priorities rather than with comprehensive vector surveillance or One Health principles. Broadening research and surveillance to include *Culex* and other understudied genera is essential for anticipating and mitigating future vector-borne disease threats in an era of rapid environmental change. One step the research community can conduct to help resolve this neglect is to simply publish the data that already exist. Often, a study focused on malaria or dengue vectors also obtains mosquito community data on additional mosquito taxa that do not get included in publications, and thus those data go “extinct” [111]. Including these data in publications, even if just in supplementary material, would help to allow those additional mosquito community data to reach the peer-reviewed literature. Journal editors and reviewers should be encouraged to allow and support additional data to be included in publications, even if tangential to the core study. In addition, public health agencies and research institutions should allocate resources to studying mosquito communities more comprehensively, and ideally proportional to the respective human and animal disease burdens [112].

This systematic review demonstrates that mosquito research in malaria- and dengue-endemic countries remains disproportionately focused on *Anopheles* and *Aedes* mosquitoes, while *Culex* vectors of several zoonotic and emerging pathogens, remain comparatively underreported. The comparatively lower reporting of *Culex* research in endemic regions may limit early detection and response to zoonotic arboviruses such as WNV, JEV, and SLEV. Broadening mosquito surveillance and research beyond the dominant human-amplified disease systems is therefore essential for integrated vector-borne disease preparedness. In an era of climate change, urbanization, and expanding vector ranges, more balanced investment in *Culex* and other understudied genera will be critical for advancing One Health surveillance and reducing the risk of future outbreaks.

## Supplementary Information

Additional file 1.

Additional file 2.

Additional file 3.

## Data Availability

All data underlying the findings are fully available without restriction. Data generated or analyzed during this study are included in this published article and its additional files.

## Abbreviations

AIC: Akaike information criterion
aOR: Adjusted odds ratio
CDC: Centers for Disease Control and Prevention
CI: Confidence interval
DHARMa: Residual diagnostics for hierarchical (multi-level/mixed) regression models (R package)
GDP: Gross domestic product
GLMM: Generalized linear mixed model
JE: Japanese encephalitis
JEV: Japanese encephalitis virus
OR: Odds ratio
SD: Standard deviation
SLEV: St. Louis encephalitis virus
UN: United Nations
WDI: World Development Indicators (World Bank)
WHO: World Health Organization
WNV: West Nile virus
ZIKV: Zika virus
