# Supplementary material for: Forgotten in the tropics: research on Culex mosquitoes is overshadowed in malaria and dengue-endemic regions
Source: Parasit Vectors. 2026 Apr 17;19:248. doi: 10.1186/s13071-026-07309-0 (PMC13267598; doi:10.1186/s13071-026-07309-0)
Supplement: Supplementary file 2 — Additional file 2. [file 13071_2026_7309_MOESM2_ESM.docx]

**Supplementary tables:**

**Table S1.** Included studies by Genus- level counts, year, and endemicity

| **Genus** | **Total Studies** | **Studies %** | **Total Countries** | **Year 2010** | **Year 2020** | **Both Endemic** | **Dengue only** | **Malaria only** | **Nonendemic** |
| --- | --- | --- | --- | --- | --- | --- | --- | --- | --- |
| *Aedes* | 718 | 51.7% | 98 | 207 | 511 | 246 | 89 | 105 | 278 |
| *Anopheles* | 691 | 49.7% | 91 | 302 | 389 | 309 | 27 | 233 | 122 |
| *Culex* | 586 | 42.2% | 88 | 214 | 372 | 159 | 40 | 93 | 294 |
| *Mansonia* | 81 | 5.8% | 33 | 38 | 43 | 31 | 11 | 28 | 11 |
| *Coquillettidia* | 52 | 3.7% | 24 | 29 | 23 | 15 | 4 | 16 | 17 |
| *Uranotaenia* | 48 | 3.5% | 24 | 21 | 27 | 8 | 7 | 18 | 15 |
| *Armigeres* | 43 | 3.1% | 14 | 14 | 29 | 24 | 7 | 1 | 11 |
| *Culiseta* | 41 | 3.0% | 16 | 19 | 22 | 1 | 0 | 10 | 30 |
| *Psorophora* | 38 | 2.7% | 10 | 22 | 16 | 9 | 2 | 7 | 20 |
| *Ochlerotatus* | 31 | 2.2% | 16 | 21 | 10 | 4 | 2 | 0 | 25 |
| *Toxorhynchites* | 23 | 1.7% | 11 | 16 | 7 | 7 | 2 | 5 | 9 |
| *Haemagogus* | 19 | 1.4% | 4 | 7 | 12 | 14 | 0 | 3 | 2 |
| *Wyeomyia* | 17 | 1.2% | 6 | 13 | 4 | 5 | 2 | 4 | 6 |
| *Orthopodomyia* | 15 | 1.1% | 6 | 9 | 6 | 4 | 0 | 1 | 10 |
| *Sabethes* | 8 | 0.6% | 3 | 4 | 4 | 6 | 0 | 1 | 1 |
| *Lutzia* | 6 | 0.4% | 5 | 2 | 4 | 1 | 0 | 4 | 1 |
| *Tripteroides* | 6 | 0.4% | 4 | 1 | 5 | 1 | 5 | 0 | 0 |
| *Mimomyia* | 5 | 0.4% | 5 | 1 | 4 | 1 | 1 | 3 | 0 |
| *Aedeomyia* | 5 | 0.4% | 5 | 2 | 3 | 2 | 1 | 1 | 1 |
| *Malaya* | 5 | 0.4% | 4 | 1 | 4 | 3 | 2 | 0 | 0 |
| *Deinocerites* | 4 | 0.3% | 3 | 2 | 2 | 0 | 0 | 2 | 2 |
| *Heizmannia* | 2 | 0.1% | 2 | 0 | 2 | 1 | 1 | 0 | 0 |
| *Topomyia* | 1 | 0.1% | 1 | 0 | 1 | 0 | 1 | 0 | 0 |
| *Shannoniana* | 1 | 0.1% | 1 | 0 | 1 | 0 | 0 | 1 | 0 |
| *Udaya* | 1 | 0.1% | 1 | 0 | 1 | 0 | 0 | 0 | 1 |

**Table S1. Included studies by genus level, year, and endemicity (inclusive).**
Counts are numbers of included studies in 2010 and 2020. “Studies %” is 100×(Total Studies)/**1,389** (total studies across both years); values may not sum to 100% due to rounding. A single study can report multiple genera, so genus totals are not mutually exclusive. “Total Countries” is the number of unique countries with ≥1 study reporting that genus. Endemicity columns are mutually exclusive categories (Both, Dengue-only, Malaria-only, Non-endemic) defined from CDC/WHO lists (Supplement S1); “Both” is the inferred overlap of malaria- and dengue-endemic lists. Year columns indicate publication year.

Table S2. Studies per country

| Country | No. of Studies | % of Studies |
| --- | --- | --- |
| United States | 178 | 12.8% |
| Brazil | 123 | 8.9% |
| India | 74 | 5.3% |
| China | 45 | 3.2% |
| Kenya | 43 | 3.1% |
| Tanzania | 39 | 2.8% |
| Argentina | 38 | 2.7% |
| Malaysia | 34 | 2.4% |
| Cameroon | 31 | 2.2% |
| Thailand | 31 | 2.2% |
| Mexico | 30 | 2.2% |
| Colombia | 29 | 2.1% |
| Burkina Faso | 27 | 1.9% |
| Indonesia | 27 | 1.9% |
| Iran | 26 | 1.9% |
| Italy | 26 | 1.9% |
| Benin | 24 | 1.7% |
| Sri Lanka | 22 | 1.6% |
| Germany | 21 | 1.5% |
| Côte d’Ivoire | 20 | 1.4% |
| South Korea | 20 | 1.4% |
| Spain | 20 | 1.4% |
| Ethiopia | 19 | 1.4% |
| Nigeria | 17 | 1.2% |
| Senegal | 17 | 1.2% |
| Mali | 16 | 1.2% |
| Ghana | 15 | 1.1% |
| Japan | 15 | 1.1% |
| Vietnam | 15 | 1.1% |
| Canada | 13 | 0.9% |
| Uganda | 13 | 0.9% |
| Cuba | 12 | 0.9% |
| France | 12 | 0.9% |
| Zambia | 12 | 0.9% |
| South Africa | 11 | 0.8% |
| Singapore | 10 | 0.7% |
| Madagascar | 9 | 0.6% |
| Congo | 8 | 0.6% |
| French Guiana | 8 | 0.6% |
| Malawi | 8 | 0.6% |
| Netherlands | 8 | 0.6% |
| Sweden | 8 | 0.6% |
| Cambodia | 7 | 0.5% |
| Laos | 7 | 0.5% |
| Pakistan | 7 | 0.5% |
| Russia | 7 | 0.5% |
| Egypt | 6 | 0.4% |
| Panama | 6 | 0.4% |
| Saudi Arabia | 6 | 0.4% |
| Sudan | 6 | 0.4% |
| Algeria | 5 | 0.4% |
| Bangladesh | 5 | 0.4% |
| Democratic Republic of the Congo | 5 | 0.4% |
| Mozambique | 5 | 0.4% |
| Trinidad and Tobago | 5 | 0.4% |
| Angola | 4 | 0.3% |
| Bhutan | 4 | 0.3% |
| Cabo Verde | 4 | 0.3% |
| Costa Rica | 4 | 0.3% |
| Croatia | 4 | 0.3% |
| Ecuador | 4 | 0.3% |
| Morocco | 4 | 0.3% |
| Myanmar | 4 | 0.3% |
| Niger | 4 | 0.3% |
| Papua New Guinea | 4 | 0.3% |
| Peru | 4 | 0.3% |
| Philippines | 4 | 0.3% |
| Portugal | 4 | 0.3% |
| Puerto Rico | 4 | 0.3% |
| Taiwan | 4 | 0.3% |
| Venezuela | 4 | 0.3% |
| Austria | 3 | 0.2% |
| Botswana | 3 | 0.2% |
| Gabon | 3 | 0.2% |
| Guinea | 3 | 0.2% |
| Hong Kong | 3 | 0.2% |
| New Caledonia | 3 | 0.2% |
| United Kingdom | 3 | 0.2% |
| Belize | 2 | 0.1% |
| Dominican Republic | 2 | 0.1% |
| Gambia | 2 | 0.1% |
| Guadeloupe | 2 | 0.1% |
| Guam | 2 | 0.1% |
| Guatemala | 2 | 0.1% |
| Guinea-Bissau | 2 | 0.1% |
| Honduras | 2 | 0.1% |
| Jamaica | 2 | 0.1% |
| Martinique | 2 | 0.1% |
| Palau | 2 | 0.1% |
| Suriname | 2 | 0.1% |
| Tunisia | 2 | 0.1% |
| Zimbabwe | 2 | 0.1% |
| Bahamas | 1 | 0.1% |
| Barbados | 1 | 0.1% |
| Belgium | 1 | 0.1% |
| Cayman Islands | 1 | 0.1% |
| Central African Republic | 1 | 0.1% |
| Chile | 1 | 0.1% |
| Cook Islands | 1 | 0.1% |
| Denmark | 1 | 0.1% |
| Djibouti | 1 | 0.1% |
| Fiji | 1 | 0.1% |
| French Polynesia | 1 | 0.1% |
| Haiti | 1 | 0.1% |
| Ireland | 1 | 0.1% |
| Mauritius | 1 | 0.1% |
| Mongolia | 1 | 0.1% |
| Nepal | 1 | 0.1% |
| Nicaragua | 1 | 0.1% |
| Paraguay | 1 | 0.1% |
| Poland | 1 | 0.1% |
| Saint Kitts and Nevis | 1 | 0.1% |
| Solomon Islands | 1 | 0.1% |
| Timor-Leste | 1 | 0.1% |
| Togo | 1 | 0.1% |
| Vanuatu | 1 | 0.1% |
| Wallis and Futuna | 1 | 0.1% |
|  |  |  |

**Footnote:** No. of Studies” is the number of included studies per country in 2010 and 2020 combined. “% of Studies” is 100×(No. of Studies)/**1,389**; percentages may not total 100% due to rounding. Country names were standardized for spelling/diacritics; entries include countries and territories as reported in the underlying publications.
