# Supplementary material for: Forgotten in the tropics: research on Culex mosquitoes is overshadowed in malaria and dengue-endemic regions
Source: Parasit Vectors. 2026 Apr 17;19:248. doi: 10.1186/s13071-026-07309-0 (PMC13267598; doi:10.1186/s13071-026-07309-0)
Supplement: Supplementary file 3 — Additional file 3. [file 13071_2026_7309_MOESM3_ESM.rtf]

# libslibrary(tidyverse)library(glmmTMB)library(emmeans)library(DHARMa)library(performance)library(readxl)library(janitor)library(countrycode)library(WDI)library(forcats)library(stringr)#  1) Read + basic clean ----------raw <- read_excel("~/Desktop/ms2_data.xlsx", sheet = "S2 – Included studies")dat <- raw %>%  clean_names() %>%  mutate(    year    = as.integer(year),    country = factor(country),    across(c(malaria_endemic, dengue_endemic, non_endemic),           ~ toupper(trimws(as.character(.))))  )# sanity: non_endemic cannot co-occur with malaria/denguestopifnot(  nrow(filter(dat,              non_endemic=="YES" &                (malaria_endemic=="YES" | dengue_endemic=="YES"))) == 0)# endemicity label + year factor (2010 = ref)dat <- dat %>%  mutate(    endemicity_cat = case_when(      malaria_endemic=="YES" & dengue_endemic=="YES" ~ "Both",      malaria_endemic=="YES" & dengue_endemic=="NO"  ~ "Malaria_only",      malaria_endemic=="NO"  & dengue_endemic=="YES" ~ "Dengue_only",      non_endemic    =="YES"                         ~ "Non_endemic",      TRUE                                            ~ NA_character_    ),    year_fac = factor(year, levels = c(2010, 2020))  ) %>%  filter(!is.na(endemicity_cat))# 2) GDP join (2010/2020) + z-score gdp <- WDI(country="all", indicator="NY.GDP.PCAP.CD", start=2010, end=2020, extra=TRUE) %>%  rename(gdp_pc = NY.GDP.PCAP.CD) %>%  filter(!is.na(iso3c), region!="Aggregates", year %in% c(2010, 2020)) %>%  select(iso3c, year, gdp_pc)dat <- dat %>%  mutate(iso3c = countrycode(as.character(country), "country.name", "iso3c", warn = FALSE)) %>%  left_join(gdp, by = c("iso3c","year")) %>%  mutate(    gdp_miss  = is.na(gdp_pc),    log_gdp   = ifelse(is.na(gdp_pc), NA_real_, log(gdp_pc + 1)),    log_gdp_z = as.numeric(scale(log_gdp))  )# 3) Genus flags (Yes/No per study) dat <- dat %>%  mutate(    vector_genus = coalesce(vector_genus, ""),    aedes     = as.integer(str_detect(vector_genus, regex("\\bAedes\\b", ignore_case=TRUE))),    anopheles = as.integer(str_detect(vector_genus, regex("\\bAnopheles\\b", ignore_case=TRUE))),    culex     = as.integer(str_detect(vector_genus, regex("\\bCulex\\b", ignore_case=TRUE)))  )# 4) Aggregate to ONE row per country×year cells <- dat %>%  filter(!gdp_miss) %>%  group_by(country, iso3c, year_fac) %>%  summarise(    total_n             = n(),    present_n_Culex     = sum(culex == 1),    present_n_Aedes     = sum(aedes == 1),    present_n_Anopheles = sum(anopheles == 1),    endemicity_cat      = first(endemicity_cat),    log_gdp_z           = first(log_gdp_z),    .groups = "drop"  ) %>%  mutate(    fail_n_Culex      = total_n - present_n_Culex,    fail_n_Aedes      = total_n - present_n_Aedes,    fail_n_Anopheles  = total_n - present_n_Anopheles,    endemicity_cat    = fct_relevel(endemicity_cat,                                    "Non_endemic","Dengue_only","Malaria_only","Both")  ) %>%  arrange(country, year_fac)# sanity: at most 2 rows per country (2010 & 2020)stopifnot(all(dplyr::count(cells, country)$n <= 2))# 5) Per-genus models (same fixed effects) # Helper: relevel endemicity for a given referencerelevel_for <- function(df, ref) {  df %>% mutate(endemicity_cat = stats::relevel(endemicity_cat, ref = ref))}# Per-genus analysis datasets with requested baselinescells_cx <- cells                                   # Culex ref = Non_endemic (already)cells_ae <- relevel_for(cells, "Dengue_only")       # Aedes ref = Dengue_onlycells_an <- relevel_for(cells, "Malaria_only")      # Anopheles ref = Malaria_only# Fit modelsmod_cx <- glmmTMB(  cbind(present_n_Culex, fail_n_Culex) ~ endemicity_cat + year_fac + log_gdp_z + (1|country),  data = cells_cx, family = betabinomial(link = "logit"))# testsimulateResiduals(fittedModel = mod_cx, n = 250, plot = TRUE)mod_ae <- glmmTMB(  cbind(present_n_Aedes, fail_n_Aedes) ~ endemicity_cat + year_fac + log_gdp_z + (1|country),  data = cells_ae, family = betabinomial(link = "logit"))#testsimulateResiduals(fittedModel = mod_ae, n = 250, plot = TRUE)# agreed fix: remove country RE for Anophelesmod_an <- glmmTMB(  cbind(present_n_Anopheles, fail_n_Anopheles) ~ endemicity_cat + year_fac + log_gdp_z,  data = cells_an, family = betabinomial(link = "logit"))cat("\n=== MODEL SUMMARIES ===\n")print(summary(mod_cx))print(summary(mod_ae))print(summary(mod_an))# 6) EMMEANS (contrasts vs each model’s baseline) emm_options(weights = "proportional")cat("\n=== Culex (ref = Non_endemic) ===\n")em_cx <- emmeans(mod_cx, ~ endemicity_cat)print(summary(contrast(em_cx, "trt.vs.ctrl", ref = "Non_endemic"),              type = "response", infer = c(TRUE, TRUE), adjust = "holm"))print(summary(em_cx, type = "response"))cat("\n=== Aedes (ref = Dengue_only) ===\n")em_ae <- emmeans(mod_ae, ~ endemicity_cat)print(summary(contrast(em_ae, "trt.vs.ctrl", ref = "Dengue_only"),              type = "response", infer = c(TRUE, TRUE), adjust = "holm"))print(summary(em_ae, type = "response"))cat("\n=== Anopheles (ref = Malaria_only) ===\n")em_an <- emmeans(mod_an, ~ endemicity_cat)print(summary(contrast(em_an, "trt.vs.ctrl", ref = "Malaria_only"),              type = "response", infer = c(TRUE, TRUE), adjust = "holm"))print(summary(em_an, type = "response"))# 7) Optional diagnostics do_diag <- TRUE  if (do_diag) {  quick_diag <- function(m, label) {    cat("\n===", label, "diagnostics ===\n")    print(performance::check_overdispersion(m))    set.seed(1)    r <- DHARMa::simulateResiduals(m, n = 500)    print(DHARMa::testUniformity(r))    print(DHARMa::testDispersion(r))    print(DHARMa::testZeroInflation(r))  }  quick_diag(mod_cx, "CULEX")  quick_diag(mod_ae, "AEDES")  quick_diag(mod_an, "ANOPHELES")}cat("\nDone.\n")# Lastly year change for aedesae <- summary(mod_ae)$coefficients$cond["year_fac2020", ]logOR <- ae["Estimate"]; SE <- ae["Std. Error"]aOR   <- exp(logOR)CI    <- exp(logOR + c(-1,1)*1.96*SE)pval  <- ae["Pr(>|z|)"]c(aOR=aOR, CI_lo=CI[1], CI_hi=CI[2], p=pval)
